# Supplementary material for: The genome sequence of a tachinid fly, Nowickia ferox (Panzer, 1809)
Source: Wellcome Open Res. 2023 Jun 23;8:275. [Version 1] doi: 10.12688/wellcomeopenres.19575.1 (PMC11196934; doi:10.12688/wellcomeopenres.19575.1)
Supplement: Peer review history

# Reviewer report: Reviewer response for version 1

**DOI:** 10.21956/wellcomeopenres.21685.r83928

**Published:** 2024-06-24

**Authors:** Phillip Shults

**Affiliations:** 1 USDA-ARS, Manhattan, USA

Here the author presents the genome of a tachinid fly, *Nowickia ferox*.

 The methods for how the specimen was sequenced and the pipelines used in genome assembly are clearly explained and align with current methods. The appropriate statistics and data are reported to assess the quality of the genome. A major positive for me was the interactive versions of the figures. The authors did a very nice job of this work.

 My only real question is about the HiC map. Why are there so many contact points across different chromosomes? 

 Here are a few additional comments to consider.

 Regarding “the Wash”, perhaps there is a different way to explain the distribution of this species.

 In table 2, chromosome 2 is not centered.

 Consider rephrasing to “…Nanodrop spectrophotometer and Qubit Fluorometer using a Qubit dsDNA High Sensitivity Assay kit”

Are sufficient details of methods and materials provided to allow replication by others?

Yes

Is the rationale for creating the dataset(s) clearly described?

Yes

Are the datasets clearly presented in a useable and accessible format?

Yes

Are the protocols appropriate and is the work technically sound?

Yes

Reviewer Expertise:

I primarily study population genetics and have experience with both short and long read sequencing. I'm also currently assembling an insect genome.

I confirm that I have read this submission and believe that I have an appropriate level of expertise to confirm that it is of an acceptable scientific standard.

# Reviewer report: Reviewer response for version 1

**DOI:** 10.21956/wellcomeopenres.21685.r83188

**Published:** 2024-05-20

**Authors:** Samuel H Church

**Affiliations:** 1 Department of Ecology and Evolutionary Biology, Yale University, New Haven, Connecticut, USA

This paper presents the genome assembly of a fly, relevant to the research goals of the Darwin Tree of Life Project. They performed a genome assembly using PacBio data, which was scaffolded using HiC chromatin conformation data and annotated using the BREAKER2 pipeline. They also assembled and curated the mitochondrial assembly using MitoHiFi. 

 I have examined the assembly results and find them to be excellent. I searched for the data in the repositories indicated and saw they were available as described. Their assembly results are in line with other publications from this initiative, including of tachinid flies by the authors.

Are sufficient details of methods and materials provided to allow replication by others?

Yes

Is the rationale for creating the dataset(s) clearly described?

Yes

Are the datasets clearly presented in a useable and accessible format?

Yes

Are the protocols appropriate and is the work technically sound?

Yes

Reviewer Expertise:

Evolutionary Biology, Bioinformatics, Comparative Genomics

I confirm that I have read this submission and believe that I have an appropriate level of expertise to confirm that it is of an acceptable scientific standard.
